# The effect of eating on the uptake of PSMA ligands in the salivary glands

**DOI:** 10.1186/s13550-021-00838-y

**Published:** 2021-09-26

**Authors:** V. Mohan, N. M. Bruin, J. B. van de Kamer, J.-J. Sonke, W. V. Vogel

**Affiliations:** 1 Department of Nuclear Medicine, The Netherlands Cancer Institute, Plesmanlaan 121, 1066 CX Amsterdam, The Netherlands; 2 Department of Radiation Oncology, The Netherlands Cancer Institute, Amsterdam, The Netherlands

**Keywords:** Salivary glands, Toxicity, PSMA, Radionuclide therapy, Stimulation

## Abstract

**Rationale:**

PSMA-directed therapy for metastatic prostate cancer is gaining adoption as a treatment option. However, accumulation of ^177^Lu/^225^Ac-PSMA in the salivary glands remains a problem, with risk of dose-limiting xerostomia and potentially severe effect on the quality of life. Gustatory stimulation is an approach that has commonly been used in radioactive iodine therapy to reduce accumulation in the salivary glands. However, based on theoretical differences in biodistribution, it was hypothesized that this could potentially lead to adverse increased toxicity for PSMA-ligand therapy. The primary objective of this work was to determine if gustatory stimulation by eating an assortment of sweet/fatty/acidic foods during the biodistribution phase of [^18^F]DCFPyl could result in a clinically relevant (> 30%) change in the uptake of the tracer in the salivary glands.

**Methods:**

10 patients who already received a whole-body [^18^F]DCFPyl PET/CT scan for evaluation of prostate cancer, underwent a repeat (intervention) PET/CT scan within a month of the first (control) scan. During the intervention scan, patients chose from an assortment of sweet/fatty/acidic foods, which they then chewed and swallowed for a period of time starting 1 min before tracer administration to 10 min thereafter. Data from both scans were analyzed by placing VOIs on the major salivary glands and segmenting them using relative thresholds.

**Results:**

A slight increase in PSMA uptake in the parotid glands was observed on the intervention scan when compared to the baseline scan (+ 7.1% SUL_mean_ and + 9.2% SUL_max_, *p* < 0.05). No significant difference in PSMA uptake in the submandibular glands was seen.

**Conclusions:**

Eating only slightly increases uptake of [^18^F]DCFPyl in the parotid glands. We nonetheless recommend refraining from gustatory stimulation during the administration and early biodistribution phase of radionuclide therapy with PSMA-ligands to reduce the risk of avoidable additional toxicity.

**Supplementary Information:**

The online version contains supplementary material available at 10.1186/s13550-021-00838-y.

## Introduction

Radioligand therapy (RLT) with prostate specific membrane antigen (PSMA) ligands has been gaining adoption as a treatment option for patients with metastatic prostate cancer. While the PSMA receptor is overexpressed in prostate cancer tissue, it is also expressed in certain normal tissues, such as the salivary glands [1–3]. As a result, PSMA-ligands tend to accumulate in these glands during therapy, although a large fraction of this has been attributed to an unknown, non-specific mechanism that is unassociated with receptor expression [4, 5]. Regardless, this uptake during therapy is undesirable, and can potentially result in radiation induced toxicity, manifesting as xerostomia (dry mouth syndrome). Xerostomia from [^177^Lu]Lu-PSMA-617 therapy is reportedly limited [6], but since it is administered over an increasing number cycles, the risk of toxicity is cumulative. In the case of alpha emitting [^225^Ac]Ac-PSMA-617 therapy, which has shown promise beyond [^177^Lu]Lu-PSMA-617 therapy in some cases [7], xerostomia is frequent and can be severe enough to warrant patients discontinuing treatment [8]. Thus, there is a growing need to develop strategies to mitigate this potentially dose-limiting toxicity, if PSMA therapy is to see widespread clinical use.

A strategy that is frequently employed in radioactive iodine (^131^I) therapy for differentiated thyroid cancer to reduce salivary gland toxicity, is gustatory stimulation [9]. Salivary gland cells express the sodium iodide symporter (NIS), which leads to accumulation of ^131^I within the gland [10]. Iodine taken up by the salivary glands is not organified as it is in the thyroid. Thus, by stimulating the secretion of saliva with a sialagogue like lemon juice, the residence or transit time of ^131^I within the gland is shortened, as it is excreted or washed out [11]. On the other hand, this stimulation also leads to an increase in blood flow to the glands [12], and given their extensively perfused vasculature, this could result in an undesirable increase in the delivery of ^131^I to the glands. This is sometimes referred to as the ‘rebound effect’, and its impact is disputed [13].

Gustatory stimulation has not been tested in the context of PSMA-ligands before. It could either increase tracer uptake due to increased perfusion, or reduce it by increasing excretion. Both potential effects on (specific and/or non-specific) PSMA uptake represent important knowledge that can contribute toward optimising guidelines for diagnostic procedures or therapy.

Positron emission tomography (PET) with diagnostic PSMA-ligands like [^68^Ga]Ga-PSMA-11 or [^18^F]DCFPyl (a radiofluorinated PSMA inhibitor) allows for screening the effects of such strategies, without the consequences that any unforeseen changes in biodistribution would have on therapy. In this study, we examined the effect of gustatory stimulation by eating during the initial administration and biodistribution phase, on the uptake of [^18^F]DCFPyl in the salivary glands using intra-patient control-intervention PET scans. A change of more than 30% of the baseline was considered clinically relevant.

## Methods

The Medical Ethics Committee of the Netherlands Cancer Institute (CCMO trial registration NL71902.031.20) approved the study protocol. The study was conducted in accordance with the Declaration of HELSINKI. Written and oral informed consent was obtained from all patients prior to study entry.

### Study population

The study included 10 patients with prostate cancer, who recently (< 1 month ago) received a [^18^F]DCFPyl PET/CT whole-body scan for evaluation of prostate cancer on clinical indication, wherein the salivary glands were clearly visible. This first scan served as the baseline or control scan. Patients with a history of disease or treatment involving the salivary glands were excluded. Patients who received any treatments since the control scan was acquired were excluded.

### Study procedure and image acquisition

On the days of both the control and intervention scans, patients were pre-hydrated with 0.5 L of water and did not consume food shortly before tracer administration. For the control scan, patients were injected intravenously with 200 MBq of [^18^F]DCFPyl (good manufacturing practice grade, produced by BV Cyclotron VU, The Netherlands). After an incubation time of 45–60 min, patients were scanned from the upper thighs to the base of the skull using a digital PET/CT scanner (Vereos, Philips Healthcare, The Netherlands). A low dose CT scan was acquired with 120 kV, 30 mAs and 2 mm slices. PET images were acquired with 2 min per bed position. All scans were reconstructed iteratively using the ordered subset estimation maximization algorithm (3 iterations and 15 subsets) to 4 × 4 × 4 mm^3^ voxels, with a gaussian filter full width-half maximum of 3 mm and with attenuation correction.

The intervention PET scan was performed within a month of the control scan. On the day of the intervention scan, patients were offered a selection of salivary stimulating food items rich in sugars, acids or fats, such as caramel, fudge, sour candy, chocolate cookies, liquorice and nougat. Patients were asked to chew and swallow the food items of their preference for a period starting 1 min prior to injection of [^18^F]DCFPyl, until 10 min after injection. The remainder of the imaging procedure for the intervention scan was identical to that of the control scan, and so were the acquisition and reconstruction parameters.

### Image analysis

Uptake on the PET scans was measured quantitatively using in-house developed software. Standardised uptake values corrected for lean body mass (SUL) were calculated using James’ formula [14]. Four 3D volumes of interest (VOI) were initially placed around each of the parotid and submandibular glands on each scan. A relative threshold of 20% of the maximum uptake value within each VOI was used to segment them. SUL_mean_ and SUL_max_ for each of the salivary glands were measured. Additionally, the liver and aorta were included as reference organs. SUL_mean_ for these organs was measured by placing a spherical VOI of 3-cm-diameter [15] in the same representative reference location in both scans.

### Statistical analysis

Statistical analysis was done using R v4.0.4 [16]. For the salivary glands, a linear mixed effects analysis of the relationship between uptake parameters and stimulation was performed using the lme4 package [17]. This was done to account for multiple measurements per gland type (left and right) from individual patients. Stimulation (control or intervention scan) and laterality (left or right) were entered as fixed effects without any interaction. Individual intercepts for each patient were fit as random effects. Log likelihood ratio tests were used to arrive at p-values, by comparing the full model to a reduced model without the stimulation fixed effect. Comparisons between the control and intervention uptake for the reference organs were done using a two-tailed paired samples t-test, after checking for normality according to the Shapiro–Wilk test. Significance level of *α* = 0.05 was selected in all tests.

## Results

### Demographics

The 10 included male patients had an average age of 66 years (range of 48–75). On the days of the intervention scan, patients selected from an assortment of saliva stimulating foods and consumed on average 116 g (range of 60–200 g) over the course of 11 min.

### PET/CT data

**Fig. 1:**
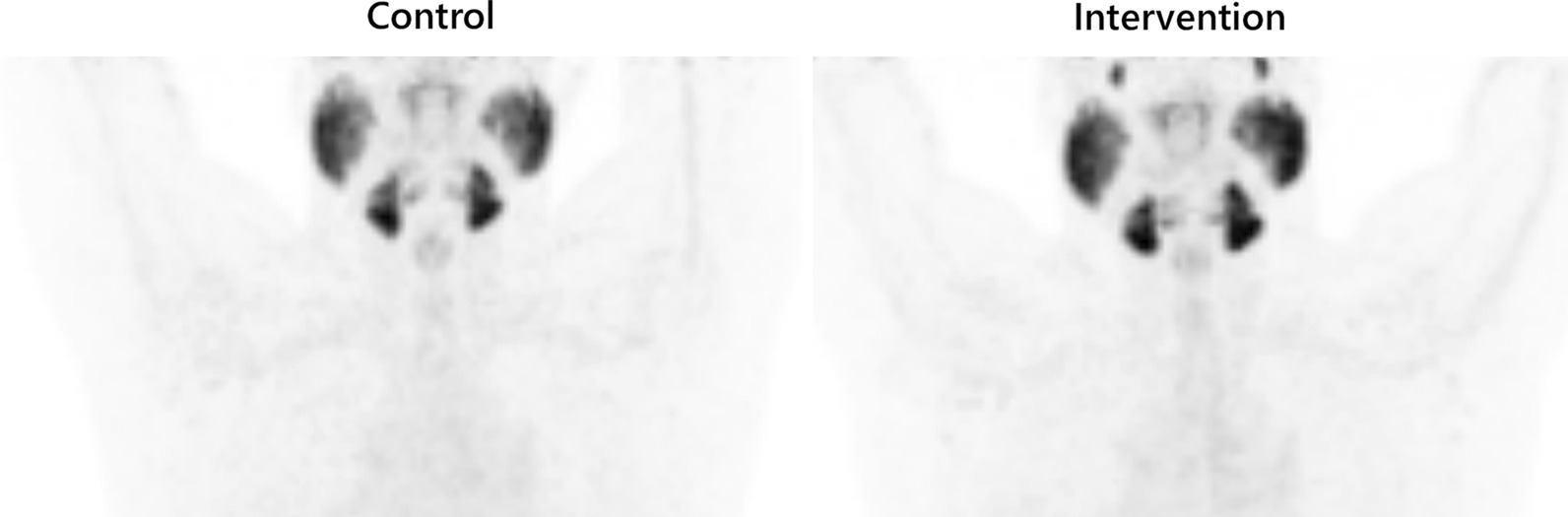
Coronal maximum intensity projections of the head and neck region from control and intervention [^18^F]DCFPyl PET scans of patient number 1. This patient demonstrated a 11.5% increase in parotid gland SUL_mean_ after gustatory stimulation

**Fig. 2:**
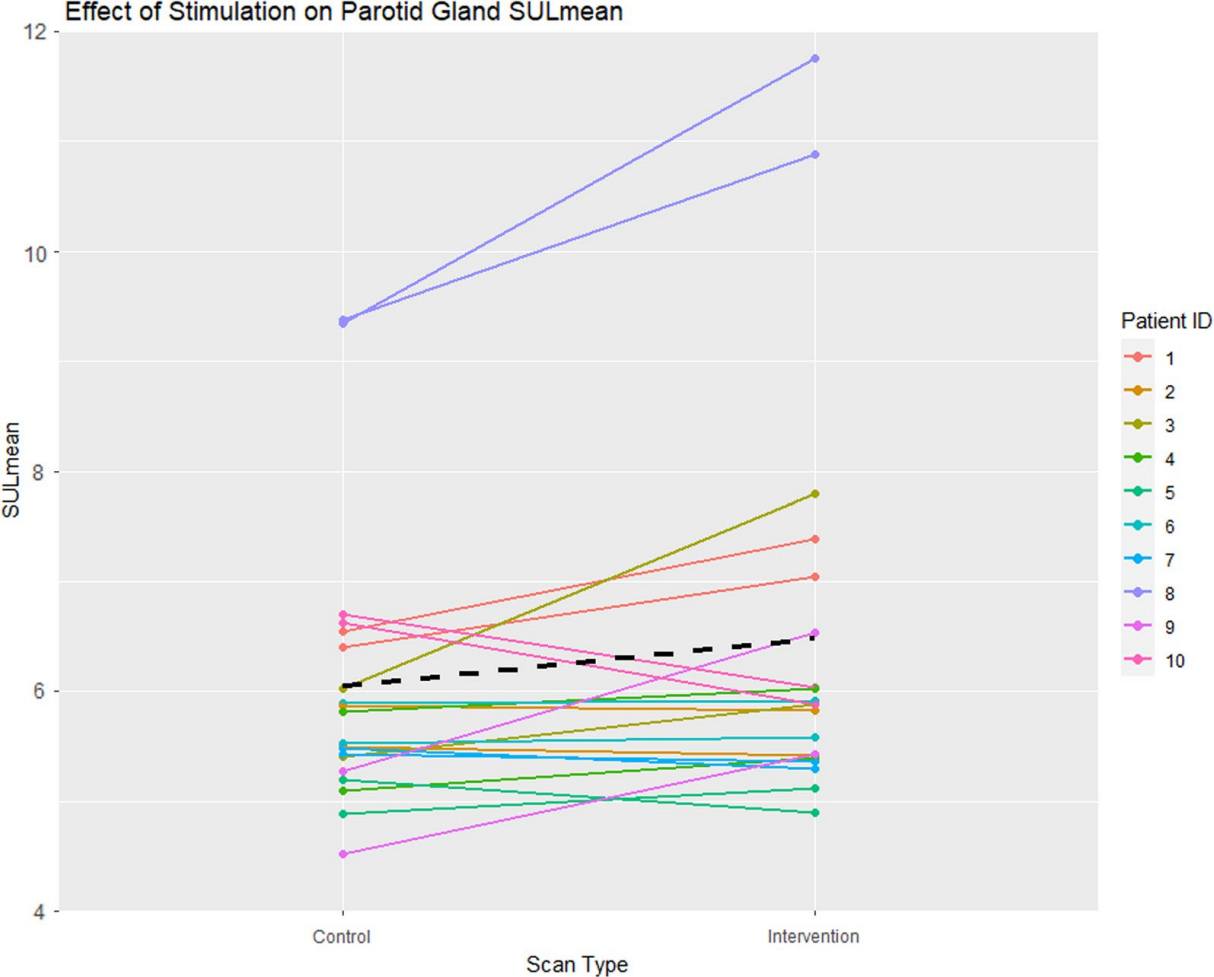
Effect of gustatory stimulation on parotid gland SUL_mean_. Each patient is represented by a unique colour. The patients have two lines, one for the right parotid and one for the left parotid. The black dashed line depicts the overall population effect for all parotid glands of all patients

Representative control and intervention scans are shown in Fig. 1. Uncropped total body scans can be seen in Additional file 1: Supplemental Fig. 1. The mean ± standard deviation of various SUL parameters for each of the different tissue types investigated in all patients, along with the relative change (and 95% confidence intervals in squared brackets) are shown in Table 1. Salivary gland data in Table 1 are averaged laterally. Upon visual inspection of the salivary gland data, there were no obvious deviations from the assumptions of normality and homoscedasticity. The reference organs also passed normality test criteria. No significant differences between the control and intervention SUL parameters were found for the submandibular glands and the liver. In the parotid glands, a small increase in SUL_mean_ (7.1%, *p* = 0.023) and SUL_max_ (9.2%, *p* = 0.016) was observed and was statistically significant when controlling for random patient effects, though this was lower than the predefined threshold for clinical relevance (Fig. 2). A small but significant reduction in SUL_mean_ in the aorta was also observed (6.0%, *p* = 0.003). Plots for other tissues and SUL paramters can be seen in Additional file 1: Supplemental Fig. 2–6.

**Table 1:** Comparison of [^18^F]DCFPyl PET parameters in tissues for control and intervention scans

Tissue	Parameter	Control	Intervention	Relative change (%)	*p* Value
Parotid glands	SUL_mean_	6.04 ± 1.29	6.47 ± 1.82	7.1 [1.1, 13.2]	0.023*
	SUL_max_	12.29 ± 2.70	13.43 ± 3.80	9.2 [1.9, 16.6]	0.016*
Submandibular glands	SUL_mean_	6.16 ± 1.62	6.28 ± 2.29	2.1 [− 5.8, 9.9]	0.606
	SUL_max_	12.76 ± 3.16	12.99 ± 4.37	1.8 [− 6.6, 10.2]	0.667
Aorta	SUL_mean_	1.14 ± 0.13	1.07 ± 0.15	− 6.0 [− 9.4, − 2.6]	0.003*
Liver	SUL_mean_	3.95 ± 0.77	3.79 ± 0.57	− 3.9 [− 15.1, 7.2]	0.446

SUL, Standardised uptake value corrected for lean body mass corrected

*p < 0.05 is considered statistically significant

## Discussion

This study evaluated the effects of gustatory stimulation by eating on the uptake of the PSMA-ligand [^18^F]DCFPyl in salivary glands and found that it resulted in a limited increase. Despite the effect size falling short of our initial definition of clinical relevance, we believe that this is sufficient to warrant a recommendation against gustatory stimulation or eating during the administration of PSMA targeted RLTs.

### Gustatory stimulation in ^131^I therapy

The effectiveness of gustatory stimulation, as well as the timing of it, in order to reduce the absorbed dose to salivary glands from ^131^I therapy is still under debate. Various studies involving non-pharmacological sialagogues like lemon juice/slices/candy, vitamin C and tasteless chewing gum have been performed using dynamic ^99m^Tc and ^123^I scintigraphy scans, as well as multiple-time-point ^124^I PET/CT scans [18]. These studies have yielded contradictory results [19], with some hypothesizing a decrease in absorbed dose due to excretion [20, 21], some an increase due to blood flow [22], and others questioning any effect whatsoever [23, 24]. Furthermore, there is contention over whether the sialagogue in question should be given immediately after administration of ^131^I, or 24 h later [19, 21]. Nonetheless, stimulation with lemon juice at either time point is recommended in guidelines for ^131^I therapy [9].

### Translation to PSMA

Unlike iodine, PSMA-ligands that are accumulated in the salivary glands stay bound. PSMA receptors are expressed constitutively in salivary gland tissue, and a portion of the uptake is bound to these receptors. PSMA is internalized in tumours [25] and if this is also true of salivary glands, we would expect stimulation to have no effect on the excretion of it out of the gland. The other portion of the uptake is believed to be bound by a non-specific mechanism unrelated to receptor expression and may or may not be displaced by stimulation [4, 5]. Since few strategies so far have been able to lower PSMA uptake selectively in the salivary glands without compromising tumour uptake [26], we determined that this approach was worth evaluating. However, as in the case of iodine, stimulation could also lead to an increase in PSMA uptake caused by an increase in blood flow.

Our study demonstrates that gustatory stimulation during the initial administration and bio-distribution phase of injected [^18^F]DCFPyl, does indeed slightly increase (7–9%) uptake in the parotid glands, when compared to a control. No significant difference was found in the submandibular glands. A possible explanation for this is that parotid glands are largely responsible for saliva production during stimulation [27]. A slight decrease (6%) in uptake was found in the aorta. Whether or not this decrease may in part be related to the increase in uptake in the parotid glands is speculative and cannot be determined from our data, but may be worth investigating in another study.

Few prior studies have tested the effects of sialagogues in the context of PSMA-ligand uptake in the salivary glands. Afshar-Oromieh et al. [28] orally administered vitamin C to patients every 30 min, 1 h after injection of [^68^ Ga]Ga-PSMA-11 and found no changes in PSMA uptake on the 3 h post injection scan when compared to unstimulated scans from a previous dataset. The lack of a reduction, in agreement with our findings, supports the hypothesis that PSMA uptake in the salivary glands, whether specific or non-specific, cannot be displaced by stimulation. The absence of any noticeable increase is explicable by the late time point at which they administered the sialagogue. Since in our study the stimulation was immediate, an increase in blood flow would result in more unbound PSMA being delivered to the salivary glands than when compared to a stimulation at 1 h post injection, where most of the PSMA is already bound. PSMA-ligand concentration in arteries decays by 80% and plateaus within 5 min of tracer administration [29]. Furthermore, given that our study showed only a small effect size, it is unsurprising they found no significant differences.

The same group, in another study [30, 31], used lemon juice 5 times per day as well as ice packs on the first day of therapy in patients receiving [^131^I]I-MIP-1095 (a radioiodinated PSMA inhibitor) therapy. They questioned its efficacy in lowering gland uptake, which was unverifiable due to the lack of a control group. Hypothetically, externally applied icepacks should cause vasoconstriction and reduce blood flow, thereby also decreasing PSMA delivery. If lemon juice doesn’t excrete PSMA out of the gland, the increase in blood flow it produces might confound or offset any protection that ice packs may offer. However, recent studies have shown that external cooling has little to no impact on PSMA uptake [32, 33].

A limitation of our study is the low number of patients, whom were all male. Moreover, the variety and intensity of stimulatory effects that different foods can elicit, and any consequential effects on PSMA uptake were not controlled for. The results obtained are also potentially dependent on the timing at which the stimulation is given. Extrapolation of our results from diagnostic scans to a therapeutic setting may not be completely robust given potential differences in uptake and biodistribution time between diagnostic and therapeutic PSMA-ligands. We recommend that our observations be validated, preferably in another centre.

Current [^177^Lu]Lu-PSMA therapy guidelines do not recommend against eating during therapy for patients, nor do they advocate for or against the use of sialagogues [34]. They also permit for up to 6 cycles of therapy with an injected activity of 7.4 GBq per cycle. Previous studies that reported on xerostomia rates were limited to fewer cycles [6]. This escalation of treatment by increasing the number of cycles delivered can only be achieved by minimizing the risk of intolerable toxicity. As toxicity can accumulate over cycles, even small deleterious effects can add up. Dose limits in external beam radiotherapy for parotid glands and submandibular glands have been established in the range of 26–39 Gy [35, 36]. The dose to the salivary glands from a 7.4 GBq cycle of [^177^Lu]Lu-PSMA-617 therapy is estimated to be around 10.2 Gy [37]. With a few of such cycles, the cumulative dose to the salivary glands can exceed these limits, and the risk of severe or irreversible xerostomia is greatly increased. Therefore, even limited increases from gustatory stimulation may elicit clinical differences in patients and should be avoided. This is especially applicable to alpha therapy with [^225^Ac]Ac-PSMA-617, wherein the dose to the glands per cycle is much higher, and for which recommended guidelines are yet to be published.

## Conclusion

In conclusion, eating during the biodistribution phase slightly increased (7–9%) uptake of [^18^F]DCFPyl in the parotid glands. We nonetheless recommend refraining from gustatory stimulation during the administration and early biodistribution phase of radionuclide therapy with PSMA-ligands to reduce the risk of avoidable additional toxicity. Moreover, we recommend that non-pharmacological interventions, such as the one investigated in this study, be tested in a diagnostic setting before application in therapy to prevent unforeseen and undesirable changes in uptake.

## Supplementary Information

**Additional file 1**. Supplementary Material.

## Data Availability

The datasets generated and analyzed for this work may be available from the corresponding author on reasonable request.

## Abbreviations

^225^Ac: Actinium-225
CT: Computed tomography
^18^F: Fluorine-18
^68^Ga: Gallium-68
^131^I: Iodine-131
^177^Lu: Lutetium-177
RLT: Radioligand therapy
NIS: Sodium iodide symporter
PET: Positron emission tomography
PSMA: Prostate-specific membrane antigen
SUL: Standardised uptake value corrected for lean body mass
^99m^Tc: Technetium-99 m
VOI: Volume of interest
