# Supplementary material for: The effect of eating on the uptake of PSMA ligands in the salivary glands
Source: EJNMMI Res. 2021 Sep 26;11:95. doi: 10.1186/s13550-021-00838-y (PMC8473516; doi:10.1186/s13550-021-00838-y)
Supplement: Supplementary file 1 — Additional file 1. Supplementary Material. [file 13550_2021_838_MOESM1_ESM.docx]

**Supplementary material**

**
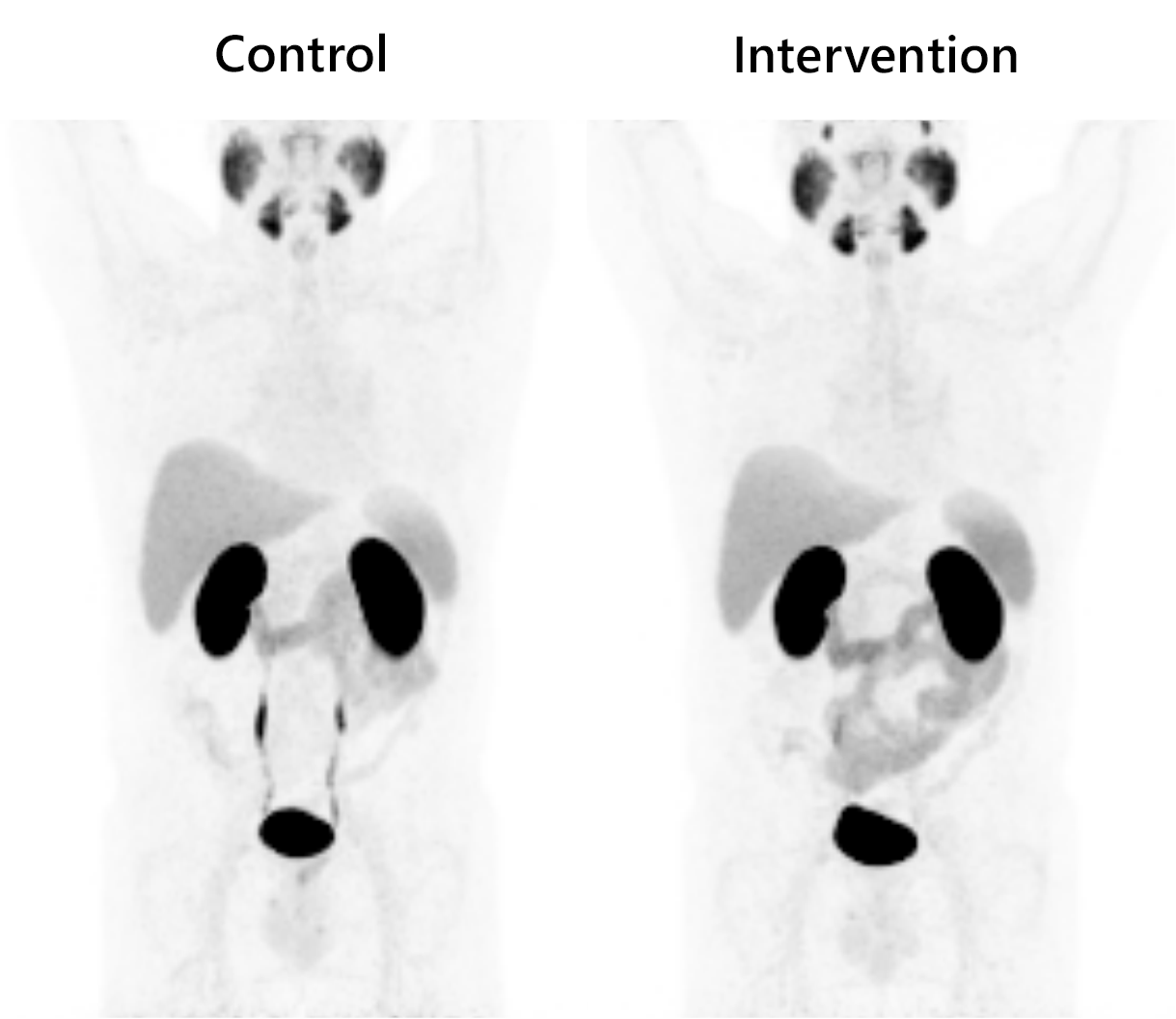
**

**Suppl. Fig. 1** Total body coronal maximum intensity projections of the control and intervention [^18^F]DCFPyl PET scans of patient number 1. All investigated tissues are clearly visible.


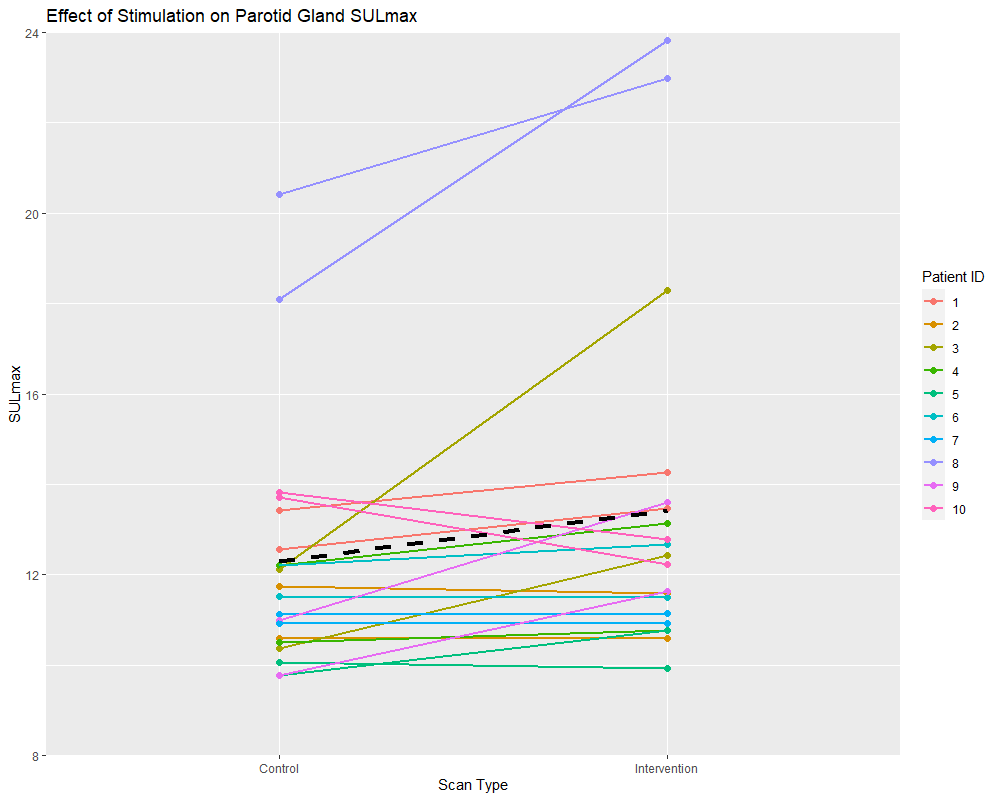
 **Suppl. Fig. 2** Effect of gustatory stimulation on parotid gland SUL_max_. Each patient is represented by a unique colour. The patients have two lines, one for the right parotid and one for the left parotid. The black dashed line depicts the overall population effect for all parotid glands of all patients.


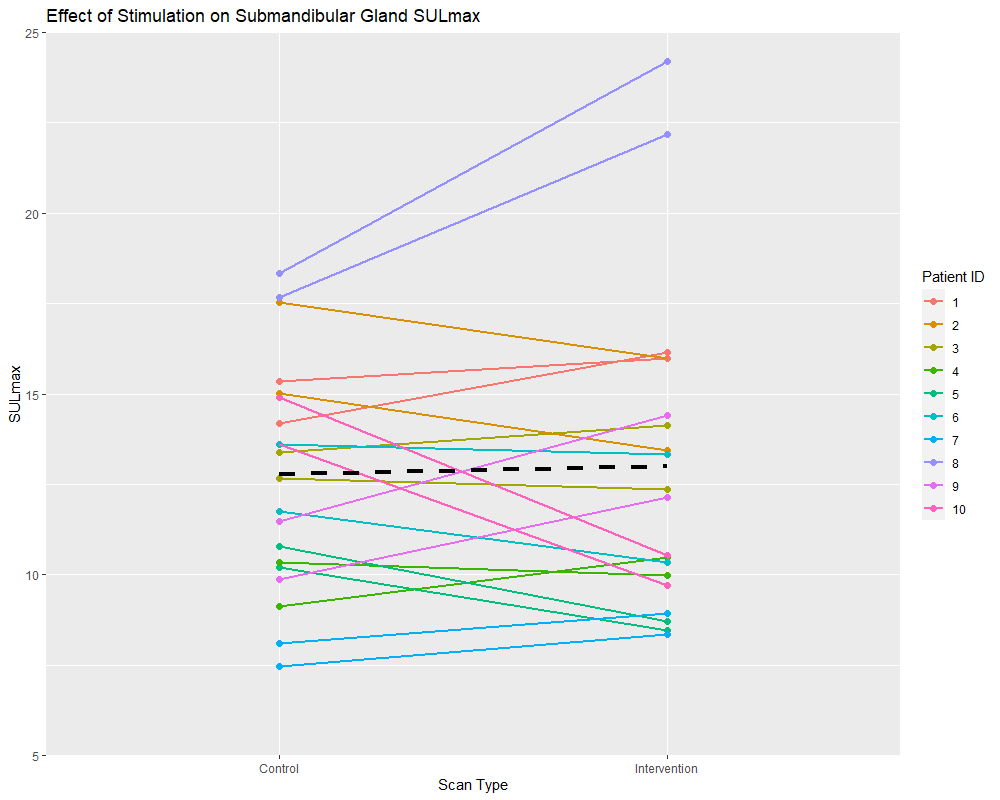
 **Suppl. Fig. 3** Effect of gustatory stimulation on submandibular gland SUL_max_. Each patient is represented by a unique colour. The patients have two lines, one for the right parotid and one for the left parotid. The black dashed line depicts the overall population effect for all submandibular glands of all patients.


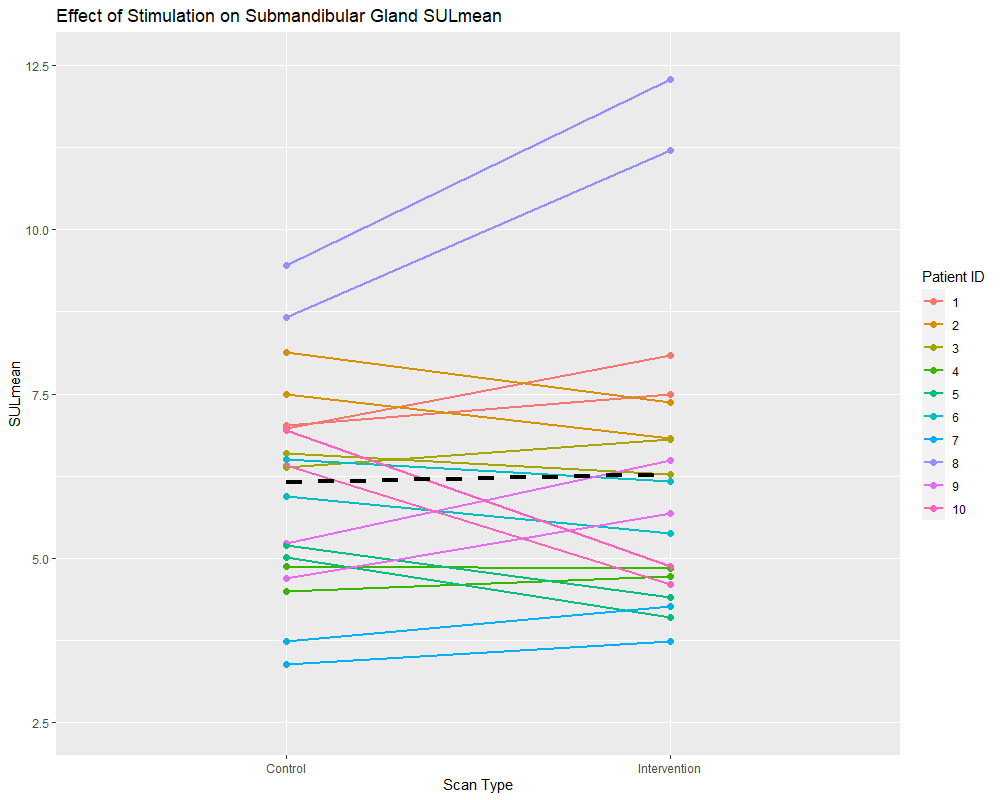


**Suppl. Fig. 4** Effect of gustatory stimulation on submandibular gland SUL_mean_. Each patient is represented by a unique colour. The patients have two lines, one for the right parotid and one for the left parotid. The black dashed line depicts the overall population effect for all submandibular glands of all patients.


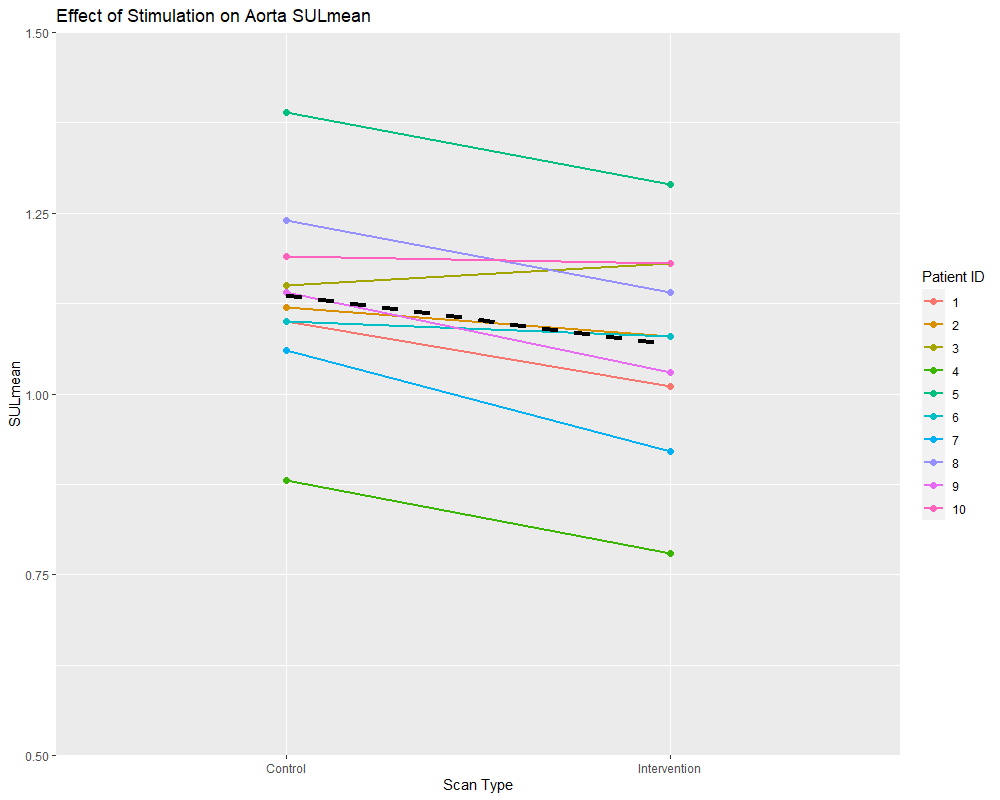
 **Suppl. Fig. 5** Effect of gustatory stimulation on aorta SUL_mean_. Each patient is represented by a unique colour. The black dashed line depicts the overall population effect.


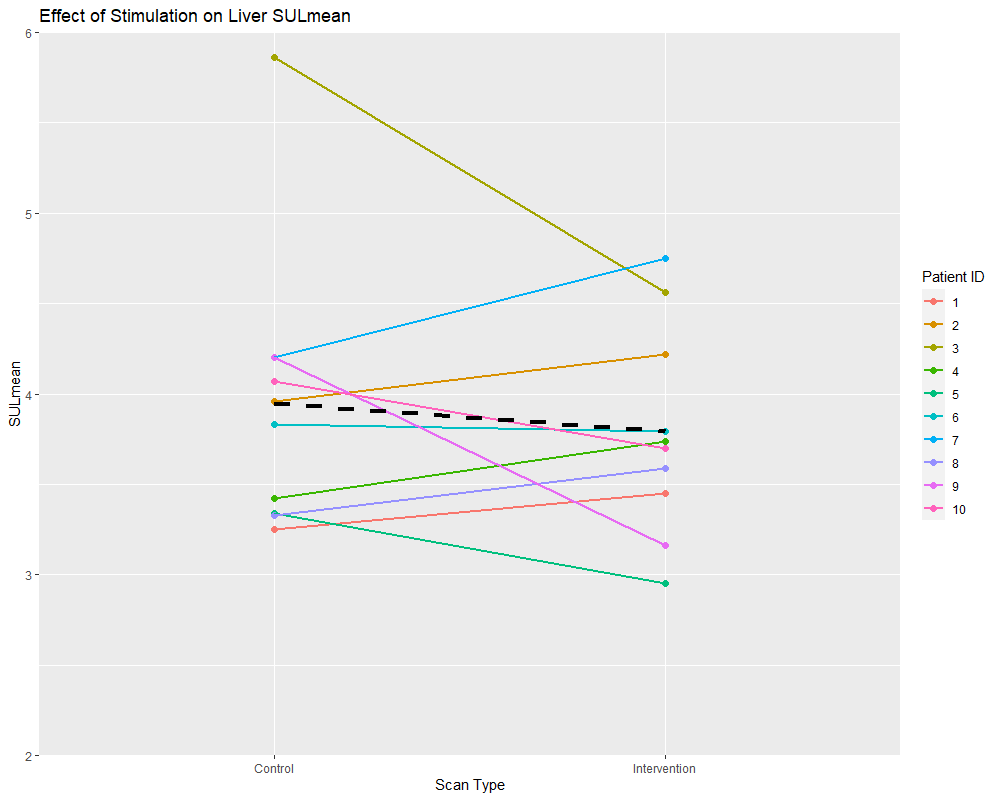
 **Suppl. Fig. 6** Effect of gustatory stimulation on liver SUL_mean_. Each patient is represented by a unique colour. The black dashed line depicts the overall population effect.
